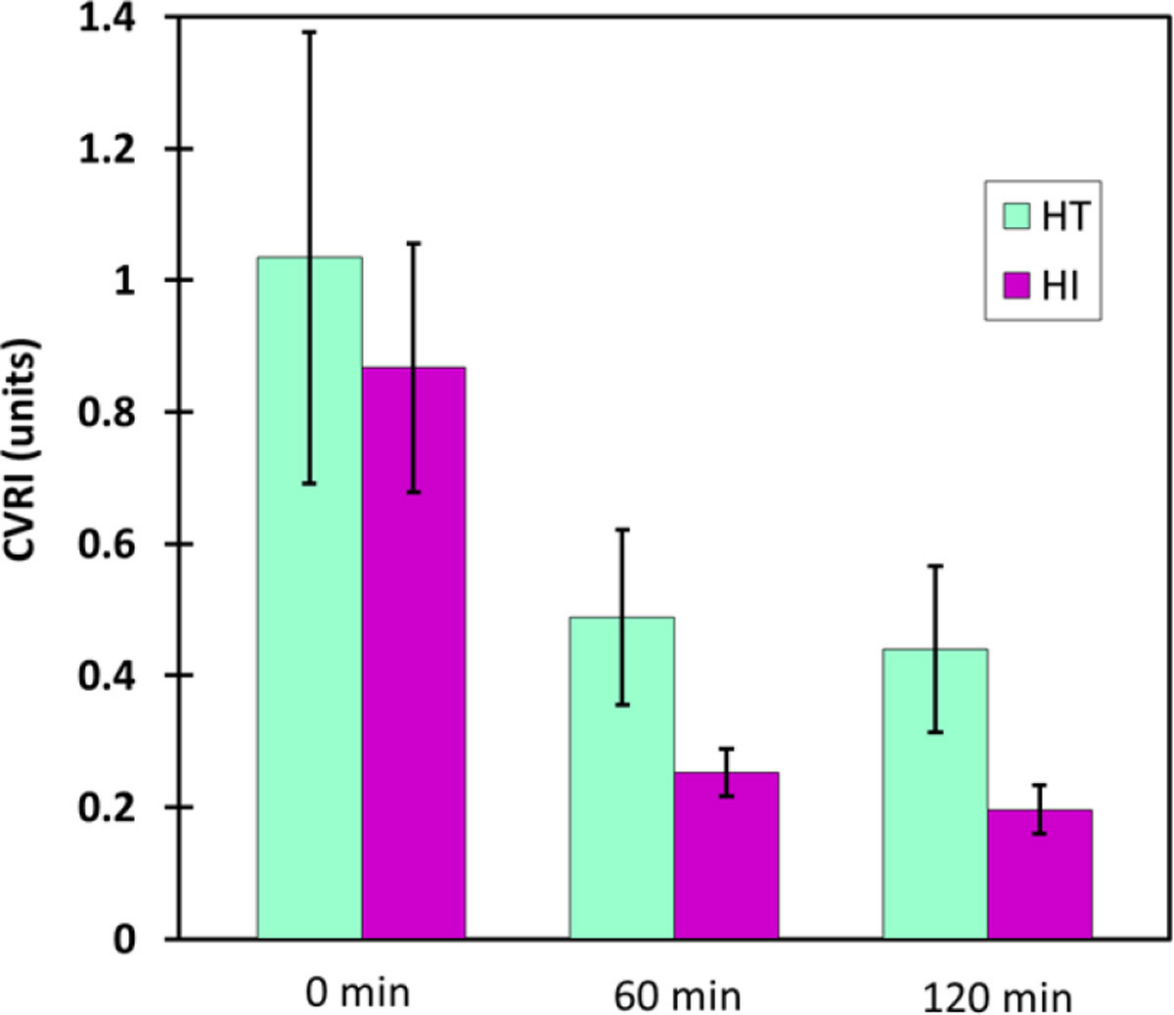# The cardiovascular reserve index (CVRI) - a surrogate index in predicting heat tolerance

**DOI:** 10.1186/2046-7648-4-S1-A158

**Published:** 2015-09-14

**Authors:** Yoram Epstein, Savyon Mazgaoker, Danit Atias, Ran Yanovich, Uri Gabbay, Yuval Heled

**Affiliations:** 1 Heller Institute of Medical Research, Sheba Medical Center, Tel Hashomer, Israel. Sackler Faculty of Medicine, Tel Aviv University, Tel Aviv, Israel. Department of Epidemiology, Beilinson Medical Centre, Petah Tiqva, Israel

## Introduction

Cardiovascular Reserve Index (CVRI) was developed as a diagnostic method for estimating quantitatively cardiovascular performance reserve [1]. It has been also been suggested in previous studies as a predictor of cardiovascular related morbidities (e.g. shock or heart failure). We aimed to investigate the CVRI ability to distinguish between heat tolerant (HT) and heat intolerant (HI) individuals during exertional heat stress.

## Methods

A modified version of the index was used in the present study as follows:

$$\text{CVRI}=100\;\cdot \;\left(\text{MAP}-\text{CVP}\right)\;\cdot \;\left(\text{H}{\text{R}}^{2}-\text{BSA}\right)$$

Where: MAP=mean arterial blood pressure (mmHg), HR=heart rate (bpm), CVP=central venous pressure (estimated as 10% of MAP) (mmHg), BSA= body surface area (m^2^). Double-blind evaluation of momentary CVRI of 15 subjects (5 HI, 10 HT) at time points 0, 60 and 120 min during a standard heat tolerance test (HTT), which consists of a moderate excise on treadmill (5 km·h^-1^, 2 % slop) in a climatic chamber (40 ºC, 40 % rh) has been performed.

## Results

Reduction in CVRI during exertional heat stress was observed in comparison to resting conditions in a comfortable climate (Figure 1). A significantly lower CVRI was found for the HI vis-à-vis the HT subjects (p < 0.0004).

## Discussion

Exercise-heat stress challenges the cardiovascular system, which is depicted by lower CVRI values. It follows that the efficiency of the subject's thermoregulatory mechanism can be characterized by the cardiovascular reserve. Thus, CVRI allows HT and HI individuals to be distinguished.

## Conclusion

The results suggest that CVRI, assessed from non-invasive measurements, can be used as a surrogate index in HTT for determining tolerance to heat even at an early stage of the test.

**Figure 1:**