# Erratum to: The cardiovascular reserve index (CVRI) - a surrogate index in predicting heat tolerance

**DOI:** 10.1186/s13728-015-0039-4

**Published:** 2015-11-12

**Authors:** Yoram Epstein, Savyon Mazgaoker, Danit Atias, Ran Yanovich, Uri Gabbay, Yuval Heled

**Affiliations:** Heller Institute of Medical Research, Sheba Medical Center, Tel Hashomer, Israel; Sackler Faculty of Medicine, Tel Aviv University, Tel Aviv, Israel; Department of Epidemiology, Beilinson Medical Centre, Petah Tiqva, Israel

## Erratum to: Extreme Physiology & Medicine 2015, 4(Suppl 1):A158 DOI 10.1186/2046-7648-4-S1-A158

The original version of this article [1] unfortunately contained an error in the equation present in the "Methods" section. The correct equation reads:

$$ {\text{CVRI}} = 100\; \cdot \;\left( {{\text{MAP}} - {\text{CVP}}} \right)/\left( {{\text{HR}}^{2}\; \cdot \;{\text{BSA}}} \right) $$
